# The 30 Years of Shifting in The Indonesian Cardiovascular Burden—Analysis of The Global Burden of Disease Study

**DOI:** 10.1007/s44197-024-00187-8

**Published:** 2024-02-07

**Authors:** Farizal Rizky Muharram, Chaq El Chaq Zamzam Multazam, Ali Mustofa, Wigaviola Socha, Andrianto, Santi Martini, Leopold Aminde, Chung Yi-Li

**Affiliations:** 1 Global Health and Social Medicine Department, Harvard Medical School, Boston, MA USA; 2 Cardiology and Respiratory Department, Imperial College London, London, UK; 3 Department of Cardiology and Vascular Medicine, Faculty of Medicine, Airlangga University, Soetomo General Hospital, Surabaya, Indonesia; 4 Faculty of Public Health, Airlangga University, Surabaya, Indonesia; 5 Population Health and Research Methods Department, School of Medicine and Dentistry, Griffith University, Brisbane, QLD Australia; 6 Institute of Public Health, National Cheng Kung University, Tainan City, Taiwan

**Keywords:** Cardiovascular disease, Preventable death, DALY, Global Burden of Disease

## Abstract

**Importance:**

Cardiovascular disease (CVD) remains the leading cause of mortality and morbidity. Compared with disease burden rates in 1990, significant reductions in Disability-Adjusted Life Years (DALYs) burden rates for CVD have been recorded. However, general DALYs rates have not changed in Indonesia in the past 30 years. Thus, assessing Indonesian CVD burdens will be an essential first step in determining primary disease interventions.

**Objective:**

To determine the national and province-level burden of CVD from 1990 to 2019 in Indonesia.

**Design, Setting, and Participants:**

A retrospective observational study was conducted using data from the Global Burden of Disease (GBD) 2019, provided by the Institute of Health Metrics and Evaluation (IHME), to analyze trends in the burden of CVD, including mortality, morbidity, and prevalence characteristics of 12 underlying CVDs.

**Exposures:**

Residence in Indonesia.

**Main Outcomes and Measures:**

Mortality, incidence, prevalence, death, and DALYs of CVD.

**Results:**

All-age CVD deaths more than doubled from 292 thousand (95% UI: 246 to 339 thousand) in 1990 and increased to 659 thousand (95% UI: 542 to 747 thousand) in 2019. All CVDs recorded increased death rates, except for rheumatic heart disease (RHD) (− 69%) and congenital heart disease (CHD) (− 37%). Based on underlying diseases, stroke and ischemic heart disease (IHD) are still the leading causes of mortality and morbidity in Indonesia, whereas stroke and peripheral artery disease (PAD) are the most prevalent CVDs. Indonesia has the second worst CVD DALYs rates compared to ASEAN countries after Laos. At provincial levels, the highest CVD DALY rates were recorded in Bangka Belitung, South Kalimantan, and Yogyakarta. In terms of DALYs rate changes, they were recorded in West Nusa Tenggara (24%), South Kalimantan (18%), and Central Java (11%). Regarding sex, only RHD, and PAD burdens were dominated by females.

**Conclusions:**

CVD mortality, morbidity, and prevalence rates increased in Indonesia from 1990 to 2019, especially for stroke and ischemic heart disease. The burden is exceptionally high, even when compared to other Southeast Asian countries and the global downward trend. GBD has many limitations. However, these data could provide policymakers with a broad view of CVD conditions in Indonesia.

**Supplementary Information:**

The online version contains supplementary material available at 10.1007/s44197-024-00187-8.

## Background

Cardiovascular Disease (CVD) remains the leading cause of mortality and morbidity worldwide. According to 2019 World Health Organization (WHO) statistics, CVD accounted for approximately 17.9 million deaths, representing 32%. CVD is also responsible for significant disability and deteriorating quality of life in affected persons, with a comparatively more substantial burden in low- and middle-income countries [1–4]. As the fourth largest nation by population, Indonesia was severely impacted by the increase in population aging, urbanization, and change in lifestyle. Previous national Studies show CVD caused by stroke and ischemic heart disease as major causes and burden at national level [5].

The Global Burden Disease (GBD) study is a collaborative multi-national research study that estimates the burden of multiple diseases and their contributing risk factors at the global, regional, and national levels. Research that analyzes the burden of cardiovascular disease has been conducted globally [6–10]. However, specific analysis assessing the CVD burden at the national level, particularly in Southeast Asia, needs to be more comprehensive. The recent GBD 2019 study revealed that Indonesia was the 6th country with the highest number of CVD deaths worldwide in 2019, with an estimated 375,479 deaths. Furthermore, CVD was responsible for the most sizeable DALY (Disability-Adjusted Life Years) compared to other diseases in Indonesia, accounting for 12.4% of total DALYs in the country [3, 11, 12].

Using data from 1990 to 2019, this study analyzes the trends in CVD's overall burden (incidence, prevalence, morbidity, and mortality) and the 11 underlying CVD conditions in Indonesia. This study will depict the differential trends in CVD burden and its attributable risk factors across Indonesia, including its provinces and neighboring regions. By doing so, it aims to provide health authorities with an assessment of the existing burden of CVD and its varying trends. This information will enable health authorities to prioritize future public health initiatives and healthcare services.

## Methods

The 2019 Global Burden of Disease study continuously provides consistent and updated global, regional, and national estimates on the burden of diseases, injuries, and risk factors by integrating all available data [13]. Briefly, the 2019 GBD study provides comprehensive and systematic assessments from 1990 to 2019 of age-specific and sex-specific mortality and years of life lost (YLLs) for 264 causes, prevalence, and years lived with disability (YLDs) for 328 diseases and injuries, and 84 risk factors in 204 countries and territories. Subnational evaluations in some countries have been introduced since the 2013 GBD study. The GBD study has continuously provided subnational analysis for 12 countries, including Indonesia [3, 11, 13, 14].

The general methodology of GBD 2019 and its improvements over previous iterations are described elsewhere [15]. The fatal and non-fatal estimates can be obtained from *vizhub.healthdata.org/gbd-compare/* and *ghdx.healthdata.org/gbd-results-tool.*

### Indonesia’s GBD Data Sources

The data used to calculate the estimated burden in Indonesia can be seen using the ‘GBD Input Source Tools.’ The complete list of input sources that informed Indonesia GBD estimates is available in Supplementary Table 1 [12]. Age- and sex-specific population counts were obtained from the *Indonesia Population and Housing Census (1961–2010).* Primary data sources for cause of death were obtained from the Basic Health Research surveys, Cause of Death Survey, Mortality Registration System Strengthening Project, and Indonesia Sample Registration Systems. GBD Data Input sources ranged from 2011 to 2018. Data from new rounds of important nationwide surveys, such as the Fifth National Health Service Survey and the Chronic Disease and Risk Factor Surveillance System from 2013 to 2014, were also updated in the 2019 GBD study.

### Disease and Measurements

We analyzed 11 diseases in CVD categories by GBD. We also add congenital heart disease as an addition, due to treatment for congenital heart disease in Indonesia being a collaboration between pediatric cardiac consultants and interventional pediatric cardiologists. Finally, we included 12 diseases related to heart disease, including aortic aneurysm, atrial fibrillation and flutter (AF), cardiomyopathy and myocarditis, congenital heart anomaly, endocarditis, hypertensive heart disease, ischemic heart disease (IHD), non-rheumatic valvular heart disease, other cardiovascular and circulatory diseases, peripheral artery disease (PAD), rheumatic heart disease (RHD), and stroke. Due to the addition of congenital data, we recalculated the data aggregation for total cardiovascular disease. This causes the data on cardiovascular disease in this paper and in the GBD tools to have differences. All cardiovascular disease in this paper includes congenital heart disease in the calculation unless stated differently later [3, 11, 13, 14].

To generate estimates of morbidity (incidence and prevalence) and mortality, the GBD collaborative uses three standardized modeling tools, including the Cause of Death Ensemble model (CODEm), DisMod-MR, and the spatiotemporal Gaussian process regression (ST-GPR). The CODEm is a well-structured framework that combines different modeling approaches to analyze cause of death data accounting for rates or cause fractions and covariates. The DisMod-MR tool uses a Bayesian meta-regression framework that exploits the relationship between disease epidemiological parameters such as incidence, prevalence, remission, and mortality to generate plausible and consistent estimates from the available data. The data used a 95% Range Uncertainty Interval (UI). DALYs are computed by summing years lived with disability (YLD) and years of life lost (YLL) to premature death. YLDs are the product of disease prevalence and a disability weight for the specific disease sequelae. At the same time, YLLs are derived from the development of a number of deaths and the remaining years expected to live at that age using a standard life expectancy. Further details of GBD analytical methods have been reported elsewhere [15, 16].

### Statistical Analysis

The primary measure used for the statistical analysis was the Estimated Annual Percentage Change (EAPC). The EAPC was calculated for each step of cardiovascular disease in every location for both genders in the dataset. The purpose was to quantify the temporal trends and observe how these measures changed annually over a specified interval [17].

For each unique combination of cause, measure, and location, EAPC was calculated. The rate for each cause, measure, and location was logarithmically transformed to linearize the trend over time. A simple linear regression model of the form ‘*y* = *α* + *βx* + *ε*’ was fitted to the transformed data, where ‘*y*’ is the natural logarithm of the rate, ‘*x*’ is the calendar year, ‘*α*’ and ‘*β*’ are the parameters estimated, and ‘*ε*’ is the error term. The EAPC was calculated as ‘EAPC = 100 * (exp(*β*) − 1),’ representing the average annual percentage change in the disease rate [17]. All statistical analyses were performed using R.

## Results

### Burden Change at the National Level

#### Deaths

All-age CVD deaths more than doubled from 292 thousand (95% UI: 246 to 339 thousand) in 1990 and increased to 659 thousand (95% UI: 542 to 747 thousand) in 2019. Based on underlying diseases, stroke and IHD remained the leading causes of CVD death in Indonesia, comprising 331 thousand (95% UI: 282 to 367 thousand) and 245 thousand (95% UI: 207 to 276 thousand) deaths, respectively, in 2019. However, the most substantial changes were observed for PAD, atrial fibrillation or flutter, and aortic aneurysm, with nearly threefold differences in the period. Furthermore, over 30 years, all 12 CVD death causes steadily increased concerning mortality numbers and rates, except for RHD and CHD, with 49% and 44% changes in CVD deaths (Table 1). Age-standardized CVD mortality rates in Indonesia exhibited a gradual increase (11.7%) from 346.7/100,000 population (95% UI: 293.2–398.3) in 1990 to 387.2/100,000 people (95% UI: 321.1–431.9) in 2019.

**Table 1:** All-age death numbers and age-standardized death rates for selected cardiovascular disease sub-categories and percentage changes by sex in Indonesia (1990–2019)

Disease sub-category	All-age deaths, no. in thousands (95% UI)			Age-standardized deaths rate (95% UI) per 100 000		
	1990	2019	Change	1990	2019	Change
Cardiovascular diseases						
Both	292 (246–339)	659 (542–747)	126%	346.7 (293.2–398.3)	387.2 (321.1–431.9)	11.7%
Female	150 (122–181)	318 (252–379)	112%	345.2 (280.9–414.3)	357.5 (286.6–420.4)	3.6%
Male	142 (114–172)	342 (262–422)	140%	346.5 (282.6–410.1)	416.8 (326.9–497.1)	20.3%
Aortic aneurysm						
Both	0.89 (0.72–1.16)	2.77 (2.01–3.88)	212%	1.16 (0.94–1.50)	1.67 (1.23–2.32)	43.8%
Female	0.35 (0.26–0.57)	0.97 (0.71–1.53)	178%	0.89 (0.66–1.36)	1.10 (0.82–1.73)	24.6%
Male	0.54 (0.40–0.76)	1.81 (1.13–2.63)	235%	1.48 (1.08–2.09)	2.38 (1.52–3.43)	61.1%
Atrial fibrillation and flutter						
Both	1.51 (1.25–1.83)	4.99 (3.90–6.56)	230%	2.82 (2.33–3.39)	4.33 (3.37–5.65)	53.5%
Female	0.98 (0.76–1.22)	3.08 (2.36–4.29)	216%	3.26 (2.52–3.99)	4.58 (3.49–6.38)	40.7%
Male	0.54 (0.41–0.68)	1.91 (1.38–2.47)	256%	2.25 (1.72–2.84)	3.95 (2.88–5.10)	75.6%
Cardiomyopathy and myocarditis						
Both	3.34 (2.61–5.05)	7.46 (5.43–10.30)	123%	3.81 (3.02–5.12)	4.57 (3.13–5.96)	19.9%
Female	1.55 (1.01–2.55)	2.98 (1.91–4.30)	92%	3.53 (2.23–5.32)	3.63 (2.15–5.26)	3.0%
Male	1.79 (1.38–2.93)	4.48 (2.92–6.96)	150%	4.06 (3.16–5.96)	5.64 (3.87–7.53)	39.1%
Congenital heart anomalies						
Both	14.02 (6.71–23.83)	7.81 (5.93–10.22)	− 44%	6.21 (2.99–10.53)	3.89 (2.92–5.12)	− 37.4%
Female	6.18 (2.66–11.44)	3.38 (2.47–4.53)	− 45%	5.61 (2.45–10.37)	3.43 (2.47–4.63)	− 38.9%
Male	7.84 (3.47–15.97)	4.43 (3.13–6.01)	− 43%	6.78 (3.01–13.77)	4.32 (3.00–5.92)	− 36.2%
Endocarditis						
Both	0.99 (0.71–1.44)	1.88 (1.27–2.84)	90%	0.79 (0.53–1.23)	0.89 (0.61–1.34)	11.8%
Female	0.54 (0.38–0.84)	0.95 (0.59–1.54)	78%	0.83 (0.56–1.33)	0.88 (0.55–1.44)	5.9%
Male	0.45 (0.29–0.80)	0.93 (0.55–1.66)	106%	0.75 (0.46–1.41)	0.89 (0.54–1.56)	17.8%
Hypertensive heart disease						
Both	23.67 (14.50–29.63)	50.62 (28.71–61.82)	114%	30.54 (19.40–38.16)	30.83 (18.44–37.08)	1.0%
Female	13.60 (8.60–18.01)	26.69 (16.03–34.71)	96%	33.62 (21.73–45.55)	30.77 (19.16–39.73)	− 8.5%
Male	10.07 (5.38–12.84)	23.93 (11.36–31.87)	138%	26.66 (14.70–33.24)	30.20 (14.85–38.68)	13.3%
Ischemic heart disease						
Both	97.86 (86.02–109.59)	245.34 (207.41–275.67)	151%	118.05 (103.39–132.74)	140.33 (119.78–154.99)	18.9%
Female	45.27 (38.66–53.35)	105.78 (86.28–124.04)	134%	107.24 (91.06–126.78)	118.28 (97.23–136.43)	10.3%
Male	52.59 (44.69–59.69)	139.57 (111.32–170.78)	165%	129.81 (110.47–147.92)	164.83 (133.63–194.18)	27.0%
Non-rheumatic valvular heart disease						
Both	0.42 (0.35–0.55)	0.90 (0.68–1.25)	111%	0.50 (0.41–0.62)	0.51 (0.39–0.69)	2.4%
Female	0.23 (0.15–0.30)	0.42 (0.30–0.56)	82%	0.52 (0.35–0.64)	0.46 (0.33–0.60)	− 12.0%
Male	0.19 (0.15–0.27)	0.47 (0.30–0.78)	147%	0.47 (0.36–0.65)	0.57 (0.37–0.93)	21.4%
Other cardiovascular and circulatory diseases						
Both	2.99 (2.49–3.69)	4.50 (3.64–5.76)	51%	2.90 (2.41–3.71)	2.47 (2.04–3.11)	− 14.7%
Female	1.59 (1.31–1.94)	2.26 (1.74–2.76)	42%	2.92 (2.45–3.79)	2.35 (1.81–2.83)	− 19.6%
Male	1.40 (1.04–1.99)	2.24 (1.64–3.39)	61%	2.89 (2.13–4.27)	2.62 (1.99–3.82)	− 9.4%
Peripheral artery disease						
Both	0.09 (0.06–0.11)	0.36 (0.24–0.53)	318%	0.13 (0.10–0.17)	0.25 (0.17–0.36)	90.2%
Female	0.03 (0.02–0.05)	0.11 (0.07–0.18)	274%	0.10 (0.07–0.15)	0.17 (0.11–0.26)	69.8%
Male	0.06 (0.04–0.08)	0.25 (0.14–0.39)	342%	0.17 (0.12–0.24)	0.35 (0.22–0.54)	109.3%
Rheumatic heart disease						
Both	2.55 (2.14–2.99)	1.30 (1.11–1.50)	− 49%	2.19 (1.80–2.64)	0.67 (0.57–0.76)	− 69.4%
Female	1.25 (1.01–1.49)	0.73 (0.60–0.88)	− 41%	1.92 (1.57–2.30)	0.72 (0.59–0.85)	− 62.7%
Male	1.30 (0.94–1.66)	0.57 (0.45–0.71)	− 56%	2.51 (1.78–3.29)	0.61 (0.49–0.74)	− 75.7%
Stroke						
Both	144.00 (128.32–159.35)	331.35 (282.00–367.07)	130%	177.57 (155.83–198.45)	196.74 (168.45–214.49)	10.8%
Female	78.57 (67.28–89.39)	170.41 (138.97–199.35)	117%	184.78 (155.29–212.70)	191.12 (157.88–220.20)	3.4%
Male	65.43 (56.06–74.71)	160.94 (127.95–194.17)	146%	168.67 (143.65–194.46)	200.41 (163.59–234.70)	18.8%

#### Prevalence and Incidence

All-age CVD prevalence increased by 120%, from 6968 thousand (95% UI: 5919–8174) in 1990 to 15,348 thousand (95% UI: 13,000–18,080) in 2019. Age-standardized prevalence rates (ASPR) increased by 6.1%, from 6781/100,000 (95% UI: 5749–7978) in 1990 to 7196.3/100,000 (95% UI: 6089.6–8495.3) in 2019. Based on sub-categories, stroke and PAD I had the highest prevalence rates, comprising 4918 thousand (95% UI: 4417–5484) with an ASPR of 2097/100,000 and 3325 (95% UI: 2850–3803) with an ASPR of 1640/100,000, respectively. This was followed by IHD, which had a prevalence rate of 3066 thousand (95% UI: 2621–3621) with an ASPR of 1495/100,000. All CVD sub-categories showed increased prevalence rates, except for congenital heart anomalies, with a 3% decrease in prevalence rates per 100,000 population. In terms of the biggest changes, endocarditis was recorded at 16%, followed by non-rheumatic valvular heart disease (15%) and cardiomyopathy and myocarditis (13%) (Table 2).

**Table 2:** All-Age Prevalence and Age-Standardized Prevalence Rates for Selected Cardiovascular Disease (CVD) Sub-categories and Their Percentage Change by Sex in Indonesia, 1990–2019

Disease Sub-Category	All-Age Prevalence, No. in Thousands (95% UI)			Age-Standardized Prevalence Rate (95% UI) per 100 000		
	1990	2019	Change	1990	2019	Change
Cardiovascular diseases						
Both	6968 (5919–8174)	15,348 (13,000–18,080)	120%	6781.0 (5749.6–7978.8)	7196.3 (6089.6–8495.3)	6.1%
Female	3634 (3087–4251)	7984 (6761–9391)	120%	6825.7 (5789.3–8010.6)	7211.8 (6104.8–8489.5)	5.7%
Male	3334 (2823–3934)	7364 (6223–8705)	121%	6727.6 (5690.4–7969.8)	7162.9 (6045.7–8509.4)	6.5%
Atrial fibrillation and flutter						
Both	653.04 (494.14–841.16)	1534.66 (1159.67–1974.52)	135%	809.06 (611.90–1036.64)	828.45 (626.16–1060.05)	2.4%
Female	325.58 (246.68–416.00)	772.41 (581.35–989.08)	137%	769.04 (581.57–983.29)	782.85 (591.24–999.41)	1.8%
Male	327.46 (249.58–420.87)	762.24 (579.38–980.24)	133%	854.55 (646.92–1096.82)	882.31 (666.82–1131.08)	3.2%
Cardiomyopathy and myocarditis						
Both	23.43 (17.21–31.42)	61.99 (45.54–83.64)	165%	33.11 (24.45–44.04)	37.44 (27.99–49.37)	13.1%
Female	6.82 (4.79–9.32)	18.38 (13.11–25.51)	170%	20.36 (14.50–27.93)	22.84 (16.34–30.69)	12.2%
Male	16.61 (11.93–22.72)	43.61 (31.61–59.89)	162%	47.75 (35.30–63.81)	54.98 (40.78–72.97)	15.1%
Congenital heart anomalies						
Both	459.49 (396.25–542.14)	486.32 (417.82–567.00)	6%	216.58 (187.36–254.50)	210.07 (179.13–247.97)	− 3.0%
Female	223.41 (192.04–264.97)	241.00 (206.74–281.02)	8%	214.24 (185.05–253.10)	211.82 (181.04–250.23)	− 1.1%
Male	236.08 (203.31–279.28)	245.32 (211.94–285.75)	4%	218.62 (189.83–256.50)	208.36 (178.45–245.19)	− 4.7%
Endocarditis						
Both	3.11 (2.54–3.76)	6.99 (5.68–8.61)	125%	3.17 (2.55–4.03)	3.68 (2.98–4.65)	16.0%
Female	1.65 (1.34–2.01)	3.68 (2.98–4.54)	123%	3.19 (2.56–4.05)	3.66 (2.96–4.61)	14.8%
Male	1.46 (1.20–1.77)	3.31 (2.69–4.09)	127%	3.16 (2.53–4.05)	3.72 (2.99–4.77)	17.6%
Hypertensive heart disease						
Both	344.15 (244.75–462.95)	837.61 (596.61–1138.43)	143%	423.22 (305.14–573.07)	451.86 (322.87–611.03)	6.8%
Female	146.77 (106.84–196.51)	363.60 (260.07–489.18)	148%	350.07 (251.06–471.10)	374.19 (267.62–499.10)	6.9%
Male	197.38 (139.38–269.39)	474.01 (339.15–650.82)	140%	505.32 (361.15–688.91)	542.44 (386.48–744.34)	7.3%
Ischemic heart disease						
Both	1325.71 (1137.67–1554.42)	3065.68 (2621.07–3621.20)	131%	1456.26 (1247.11–1698.30)	1495.01 (1278.73–1765.29)	2.7%
Female	476.50 (403.37–565.96)	1098.88 (926.22–1319.68)	131%	1030.01 (871.97–1221.75)	1050.65 (883.50–1252.90)	2.0%
Male	849.21 (731.46–992.01)	1966.80 (1687.01–2312.74)	132%	1931.08 (1670.43–2251.37)	2000.26 (1723.72–2347.10)	3.6%
Non-rheumatic valvular heart disease						
Both	48.50 (43.72–53.25)	124.38 (113.10–136.30)	156%	48.09 (43.82–52.39)	55.34 (50.80–60.29)	15.1%
Female	28.12 (25.35–30.95)	69.11 (62.82–75.78)	146%	54.42 (49.53–59.66)	59.80 (54.76–65.30)	9.9%
Male	20.38 (18.20–22.51)	55.26 (49.80–60.75)	171%	41.00 (37.12–45.05)	50.17 (45.75–54.83)	22.4%
Other cardiovascular and circulatory diseases						
Both	323.85 (241.33–430.76)	592.16 (444.63–788.22)	83%	214.13 (163.59–278.38)	228.24 (174.37–296.17)	6.6%
Female	165.74 (121.24–225.30)	301.94 (222.71–412.79)	82%	214.53 (161.21–285.62)	225.39 (170.31–300.48)	5.1%
Male	158.11 (117.86–208.85)	290.22 (218.57–377.80)	84%	215.23 (165.11–276.24)	233.97 (180.19–298.70)	8.7%
Peripheral artery disease						
Both	1300.89 (1110.35–1490.53)	3324.59 (2850.20–3802.88)	156%	1461.32 (1264.64–1666.71)	1640.10 (1423.77–1870.95)	12.2%
Female	880.57 (753.43–1007.28)	2259.94 (1942.98–2581.92)	157%	1889.68 (1631.38–2155.94)	2108.22 (1825.43–2408.39)	11.6%
Male	420.32 (357.00–482.13)	1064.66 (908.04–1217.20)	153%	974.87 (844.38–1115.16)	1089.73 (944.08–1240.34)	11.8%
Rheumatic heart disease						
Both	225.38 (185.58–272.49)	395.03 (328.44–475.61)	75%	135.80 (115.06–159.91)	148.85 (124.74–177.75)	9.6%
Female	126.26 (102.76–153.74)	219.43 (180.28–265.56)	74%	144.25 (121.03–171.57)	163.41 (134.78–197.24)	13.3%
Male	99.12 (82.15–119.29)	175.60 (147.05–209.80)	77%	128.32 (109.98–149.73)	136.31 (115.99–160.94)	6.2%
Stroke						
Both	2260.36 (2045.21–2490.94)	4918.49 (4417.11–5483.59)	118%	1980.22 (1784.01–2210.83)	2097.22 (1878.06–2351.78)	5.9%
Female	1252.80 (1128.70–1379.08)	2635.41 (2361.29–2945.46)	110%	2135.93 (1919.47–2376.57)	2208.94 (1976.80–2481.18)	3.4%
Male	1007.56 (911.21–1115.23)	2283.08 (2047.49–2546.13)	127%	1807.75 (1627.67–2022.15)	1960.61 (1760.43–2209.14)	8.5%

All-age Incidence increased by 90% from 799 thousand (95% UI: 669–957) in 1990 to 1521 thousand (95% UI: 1298–1786) in 2019. Age-standardized incidence rates increased by 2.8%, from 6822/100,000 (95% UI: 582.3–800.2) in 1990 to 701.3/100,000 (95% UI: 600.9–821.7) in 2019. Based on sub-categories, stroke had the highest all-age incidence in 2019, with 339.30 thousand (95% UI: 292.60–389.85) incidence. Stroke also had the highest age-standardized incidence rate, with 293.33/100.000 (95% UI: 262.25–331.60) in 2019. In terms of the biggest changes in age-standardized incidence rate, endocarditis was recorded at 30.2%, followed by non-rheumatic valvular heart disease (13.6%) and peripheral artery disease (9.2%) (Table 3).

**Table 3:** All-Age Incidence and Age-Standardized Incidence Rates for Selected Cardiovascular Disease (CVD) Sub-categories and Their Percentage Change by Sex in Indonesia, 1990–2019

Disease Sub-Category	All-Age Incidence, No. in Thousands (95% UI)			Age-Standardized Incidence Rate (95% UI), per 100 000		
	1990	2019	Change	1990	2019	Change
Cardiovascular diseases						
Both	799 (669–957)	1521 (1298–1786)	90%	682.2 (582.3–800.2)	701.3 (600.9–821.7)	2.8%
Female	404 (340–482)	781 (667–915)	93%	686.1 (586.0–803.6)	703.1 (600.4–823.7)	2.5%
Male	394 (328–476)	740 (629–872)	88%	676.4 (576.0–795.5)	696.2 (595.6–817.6)	2.9%
Atrial fibrillation and flutter						
Both	61.25 (46.16–78.70)	142.15 (107.05–182.36)	132%	65.22 (49.53–83.02)	66.74 (50.67–85.21)	2.3%
Female	30.44 (23.03–39.06)	70.35 (52.85–89.97)	131%	62.76 (47.49–80.12)	63.76 (48.21–81.25)	1.6%
Male	30.81 (23.19–39.56)	71.81 (53.80–92.64)	133%	67.83 (51.75–86.32)	69.90 (53.16–89.55)	3.0%
Cardiomyopathy and myocarditis						
Both	23.59 (18.78–29.00)	37.93 (30.24–46.67)	61%	15.97 (12.94–19.43)	15.99 (12.95–19.44)	0.1%
Female	9.54 (7.60–11.80)	15.49 (12.39–19.16)	62%	13.02 (10.51–15.96)	13.02 (10.51–15.96)	0.0%
Male	14.05 (11.13–17.32)	22.44 (17.82–27.46)	60%	19.09 (15.56–23.10)	19.09 (15.56–23.10)	0.0%
Congenital heart anomalies						
Both	164.01 (122.43–221.37)	121.87 (90.99–165.79)	− 26%	71.78 (53.58–96.89)	66.15 (49.39–89.98)	− 7.9%
Female	72.71 (54.54–98.25)	56.00 (41.57–75.56)	− 23%	65.43 (49.07–88.41)	62.58 (46.46–84.44)	− 4.3%
Male	91.31 (68.33–124.02)	65.87 (48.90–89.82)	− 28%	77.81 (58.23–105.69)	69.51 (51.60–94.78)	− 10.7%
Endocarditis						
Both	10.72 (8.57–13.15)	22.62 (18.43–27.10)	111%	7.27 (6.04–8.62)	9.47 (7.89–11.21)	30.2%
Female	5.26 (4.21–6.44)	11.10 (9.02–13.42)	111%	7.02 (5.81–8.32)	9.20 (7.64–10.95)	31.0%
Male	5.46 (4.35–6.70)	11.52 (9.37–13.86)	111%	7.53 (6.27–8.96)	9.73 (8.14–11.57)	29.3%
Hypertensive heart disease						
Both	N/A	N/A	N/A	N/A	N/A	N/A
Female	N/A	N/A	N/A	N/A	N/A	N/A
Male	N/A	N/A	N/A	N/A	N/A	N/A
Ischemic heart disease						
Both	91.76 (78.23–106.07)	178.37 (155.17–202.47)	94%	97.81 (83.97–112.38)	84.48 (74.16–95.31)	− 13.6%
Female	28.68 (24.26–33.38)	49.06 (42.34–55.80)	71%	62.89 (53.35–72.75)	48.11 (41.88–54.94)	− 23.5%
Male	63.08 (53.95–72.76)	129.31 (112.49–147.08)	105%	135.94 (117.02–155.98)	124.49 (109.23–140.73)	− 8.4%
Non-rheumatic valvular heart disease						
Both	3.85 (3.59–4.12)	9.31 (8.65–10.01)	142%	3.15 (2.95–3.37)	3.58 (3.35–3.83)	13.6%
Female	2.18 (2.02–2.34)	4.95 (4.60–5.33)	128%	3.48 (3.25–3.73)	3.78 (3.53–4.05)	8.4%
Male	1.68 (1.55–1.80)	4.36 (4.03–4.70)	160%	2.80 (2.63–3.00)	3.39 (3.17–3.62)	20.9%
Other cardiovascular and circulatory diseases						
Both	N/A	N/A	N/A	N/A	N/A	N/A
Female	N/A	N/A	N/A	N/A	N/A	N/A
Male	N/A	N/A	N/A	N/A	N/A	N/A
Peripheral artery disease						
Both	136.50 (117.31–156.34)	339.30 (292.60–389.85)	149%	138.61 (120.47–158.01)	151.31 (131.87–172.72)	9.2%
Female	91.58 (78.79–104.88)	226.38 (195.60–260.30)	147%	178.37 (154.67–202.91)	193.64 (167.97–221.52)	8.6%
Male	44.92 (38.53–51.94)	112.92 (97.29–130.20)	151%	95.16 (82.36–108.73)	104.95 (91.12–119.65)	10.3%
Rheumatic heart disease						
Both	18.94 (15.27–22.94)	26.62 (21.75–32.05)	41%	10.12 (8.47–11.94)	10.26 (8.34–12.43)	1.4%
Female	10.35 (8.21–12.65)	14.26 (11.44–17.37)	38%	10.49 (8.67–12.53)	11.07 (8.84–13.53)	5.5%
Male	8.59 (7.03–10.37)	12.36 (10.24–14.78)	44%	9.83 (8.32–11.46)	9.57 (7.92–11.43)	− 2.6%
Stroke						
Both	288.25 (258.30–325.57)	642.94 (573.04–729.35)	123%	272.23 (244.40–306.56)	293.33 (262.25–331.60)	7.8%
Female	153.75 (137.81–173.56)	333.87 (297.00–378.55)	117%	282.63 (253.16–318.85)	297.91 (265.33–337.11)	5.4%
Male	134.50 (120.22–151.90)	309.07 (275.32–351.40)	130%	260.43 (233.86–292.28)	285.59 (255.66–323.18)	9.7%

#### DALYs

**Fig. 1:**
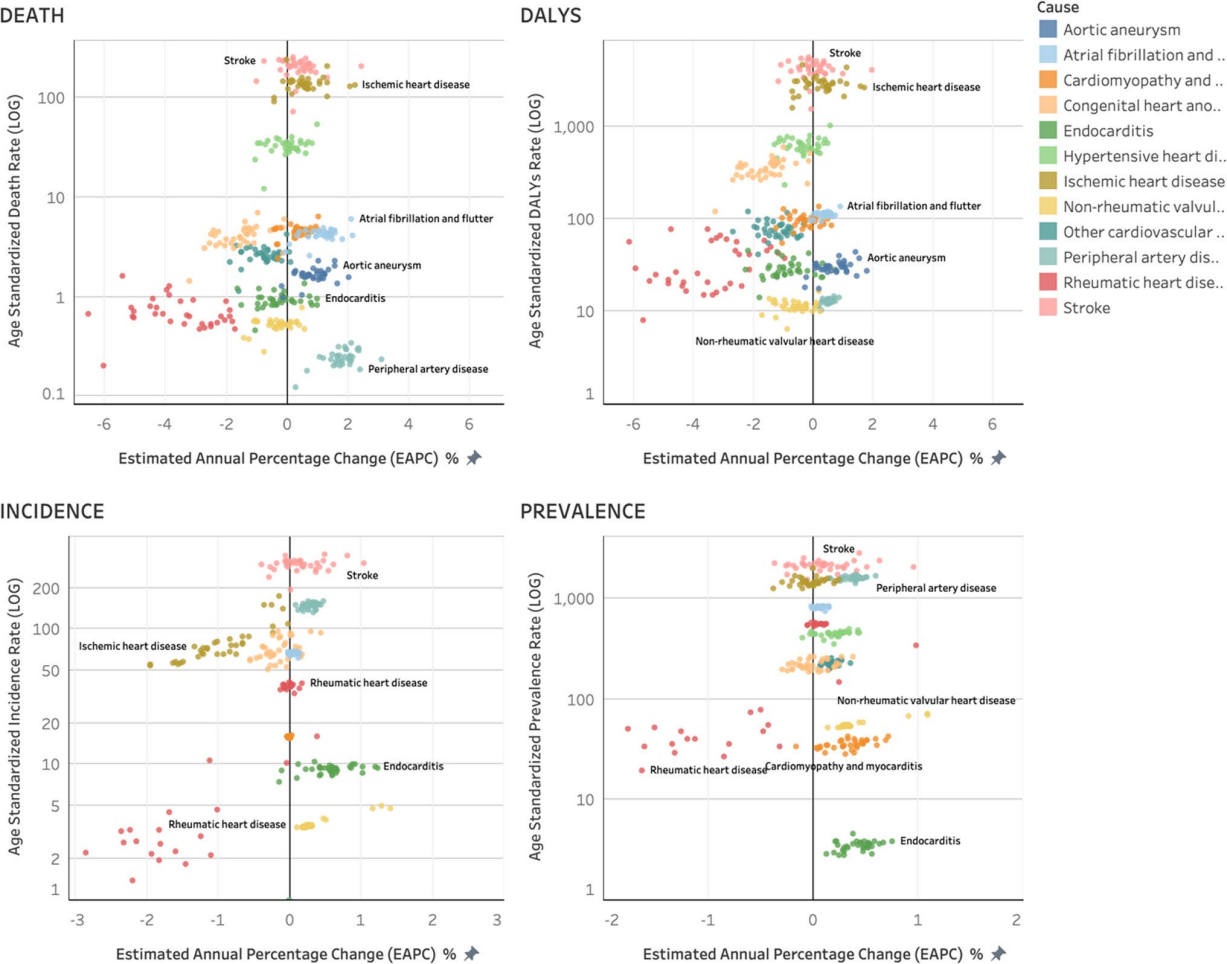
Scatter plot of DALYs rate and its change for all cardiovascular diseases Indonesia. [The graphs show four metrics of GBD: Death, DALYs, incidence, and prevalence. *x*-axis shows the estimated percentage change (EAPC), whereas *y*-axis shows age-standardized DALYs rate in 2019 on the logarithmic scale. Each dot represents a province in Indonesia, and the color indicates different cardiovascular diseases.]

The number of DALYs increased by 86% between 1990 and 2019, while DALYs rates/100,000 population remained relatively stable with only a 0.7% decrease. With an age-standardized rate (ASR) of 4021/100,000, stroke emerged as the leading cause of DALYs, while the DALY ASR for stroke remained unchanged (0%). The second largest contributor was IHD, with a DALY ASR of 2809/100,000. The rate of IHD DALYs increased by 10%. Other CVDs with higher DALY rates included PAD (19%), aortic aneurysm (237%), and atrial fibrillation and flutter (18%). The number of DALYs for CVD increased across all age groups except for RHD (Table 4; Fig. 1).

**Table 4:** All-age DALYs numbers and age-standardized DALYs rates for selected cardiovascular disease sub-categories and percentage changes by sex in Indonesia, 1990–2019

Disease Sub-Category	All-Age DALYs, No. in Thousands (95% UI)			Age-Standardized DALYs Rate (95% UI) per 100 000		
	1990	2019	Change	1990	2019	Change
Cardiovascular diseases						
Both	9257 (7547–11,181)	17,183 (14,038–19,949)	86%	8173.3 (6883.8–9534.0)	8115.7 (6715.2–9254.7)	− 0.7%
Female	4569 (3631–5735)	7719 (6087–9435)	69%	7973.1 (6509.7–9656.2)	7184.5 (5721.4–8621.7)	− 9.9%
Male	4687 (3627–6056)	9464 (7233–11,894)	102%	8358.8 (6738.5–10,190.4)	9060.5 (7072.8–11,137.6)	8.4%
Aortic aneurysm						
Both	21.5 (17.2–28.9)	61.2 (43.1–86.3)	184%	22.0 (17.7–28.9)	30.0 (21.6–42.0)	36.7%
Female	8.1 (5.9–14.2)	19.9 (14.3–32.3)	147%	16.1 (12.0–26.4)	18.9 (13.7–30.4)	17.3%
Male	13.5 (9.6–19.1)	41.3 (25.2–61.0)	206%	28.4 (20.7–40.2)	42.8 (26.7–62.2)	50.9%
Atrial fibrillation and flutter						
Both	74.6 (54.8–98.5)	193.0 (143.4–249.1)	159%	96.3 (72.4–126.8)	113.2 (86.6–143.7)	17.5%
Female	40.2 (29.6–52.2)	103.1 (77.6–134.2)	157%	98.8 (74.4–125.9)	112.5 (86.7–144.5)	13.8%
Male	34.4 (24.4–46.9)	89.9 (63.8–119.2)	161%	93.1 (68.3–124.7)	113.5 (84.2–146.6)	21.9%
Cardiomyopathy and myocarditis						
Both	121.0 (84.3–226.2)	201.1 (141.7–299.5)	66%	94.0 (72.1–147.2)	93.9 (68.5–130.0)	− 0.1%
Female	54.0 (32.0–103.7)	72.0 (46.4–112.0)	33%	82.1 (53.2–142.6)	68.4 (45.2–101.6)	− 16.7%
Male	67.0 (47.5–129.4)	129.1 (83.4–210.7)	93%	106.3 (81.9–178.8)	121.5 (83.3–184.3)	14.3%
Congenital heart anomalies						
Both	1224.2 (583.5–2071.8)	671.6 (508.7–884.2)	− 45%	540.1 (258.0–913.0)	338.1 (253.4–450.0)	− 37.4%
Female	537.2 (231.9–990.5)	289.2 (208.4–388.3)	− 46%	485.4 (210.1–894.5)	297.6 (212.5–401.5)	− 38.7%
Male	687.0 (301.5–1397.5)	382.3 (269.2–520.0)	− 44%	592.0 (260.9–1202.9)	376.4 (260.2–514.2)	− 36.4%
Endocarditis						
Both	45.9 (35.0–67.0)	67.4 (46.0–100.3)	47%	28.9 (21.3–42.1)	26.7 (18.4–39.3)	− 7.6%
Female	25.0 (16.4–40.1)	32.8 (20.2–52.9)	31%	30.6 (21.5–47.6)	26.1 (16.3–42.1)	− 14.8%
Male	20.9 (14.6–33.8)	34.6 (20.7–62.5)	65%	27.1 (18.0–46.8)	27.3 (16.6–48.0)	0.5%
Hypertensive heart disease						
Both	593.7 (365.6–744.8)	1162.9 (649.5–1428.8)	96%	620.5 (396.2–773.4)	575.8 (334.8–693.7)	− 7.2%
Female	326.1 (201.5–426.1)	561.1 (326.8–736.3)	72%	661.1 (419.2–863.9)	541.6 (324.3–695.5)	− 18.1%
Male	267.6 (143.5–339.7)	601.8 (290.2–807.4)	125%	572.2 (314.2–720.1)	605.5 (308.4–791.1)	5.8%
Ischemic heart disease						
Both	2693 (2374–3014)	6140 (5129–7098)	128%	2553.2 (2257.0–2847.3)	2809.4 (2379.6–3182.4)	10.0%
Female	1169 (1000–1371)	2373 (1909–2904)	103%	2203.8 (1888.7–2576.2)	2179.6 (1770.9–2590.8)	− 1.1%
Male	1524 (1299–1722)	3767 (2998–4711)	147%	2922.1 (2499.8–3296.9)	3473.4 (2800.4–4241.5)	18.9%
Non-rheumatic valvular heart disease						
Both	12.8 (10.2–16.7)	23.7 (18.3–33.1)	85%	11.5 (9.5–14.7)	10.9 (8.6–15.0)	− 5.1%
Female	7.0 (4.4–9.5)	10.8 (7.9–14.2)	53%	12.1 (8.0–15.6)	9.7 (12.6–7.2)	− 19.8%
Male	5.8 (4.5–8.1)	12.9 (8.4–21.3)	123%	10.9 (8.4–15.1)	12.3 (8.1–19.7)	12.9%
Other cardiovascular and circulatory diseases						
Both	140.6 (116.5–171.3)	166.5 (137.1–205.9)	18%	95.2 (80.1–115.9)	70.9 (59.2–86.6)	− 25.5%
Female	74.6 (57.7–95.5)	81.4 (65.6–99.2)	9%	98.3 (79.8–119.3)	67.9 (54.7–81.8)	− 30.9%
Male	66.0 (51.3–87.9)	85.0 (63.7–121.3)	29%	92.3 (71.2–126.2)	74.4 (56.9–106.2)	− 19.5%
Peripheral artery disease						
Both	8.5 (4.9–13.8)	23.1 (13.7–36.3)	171%	11.0 (6.2–18.1)	13.0 (7.8–20.5)	18.7%
Female	5.2 (2.7–8.8)	13.0 (6.9–21.9)	150%	12.8 (6.8–22.3)	13.9 (7.6–23.7)	8.0%
Male	3.3 (2.2–5.1)	10.1 (6.4–14.8)	203%	8.7 (22.3–13.4)	11.7 (7.5–17.4)	34.7%
Rheumatic heart disease						
Both	124.5 (102.0–144.8)	65.0 (55.1–76.5)	− 48%	79.0 (66.9–92.0)	26.1 (22.2–30.5)	− 67.0%
Female	66.8 (50.2–81.1)	36.7 (30.3–44.5)	− 45%	78.0 (62.9–93.1)	29.0 (24.1–34.9)	− 62.8%
Male	57.7 (42.7–73.9)	28.3 (22.9–35.0)	− 51%	80.9 (59.0–103.1)	23.2 (18.9–28.4)	− 71.4%
Stroke						
Both	4196.4 (3799.5–4583.3)	8407.2 (7152.1–9450.9)	100%	4021.7 (3626.5–4414.7)	4007.6 (3454.6–4420.8)	− 0.4%
Female	2256.3 (1999.5–2542.5)	4125.6 (3373.4–4895.2)	83%	4194.0 (3673.2–4728.6)	3819.4 (3158.0–4462.4)	− 8.9%
Male	1940.1 (1686.6–2193.3)	4281.7 (3381.1–5209.0)	121%	3824.8 (3330.6–4322.1)	4178.7 (3401.7–4978.1)	9.3%

### Gender on Cardiovascular Disease Burden

Based on gender, we observed ASPR changes in men (6.5%) and women (5.7%). Stroke had a higher ASPR in women when compared with men (2208 vs. 1906/100,000). However, the ASPR for stroke showed higher changes in males when compared with females (8.5% vs. 3.4%). No significant differences in ASPR were recorded for PAD in men (12%) and women (12%). For IHD, ASPR was higher in males (2000/100,000) when compared with females (1050/100,000).

However, women and men experienced different changes in the last 30 years, accounting for 3.6% for women and 20.3% for men. Age-standardized CVD mortality rates in men outnumbered women across seven diseases, including stroke, IHD, congenital heart anomalies, cardiomyopathy and myocarditis, endocarditis, aortic aneurysm, and non-rheumatic valvular heart disease.

We observed significant differences in DALYs between genders, with almost all CVDs experiencing increased DALYs in males, except for three CVDs that experienced decreased DALYs in males, including RHD (− 71.4%), congenital heart anomalies (− 36.4%), and other cardiovascular and circulatory diseases (− 19.5%). These data were inversely proportional to DALYs ASRs in females. Only three CVDs showed an increased DALYs ASR: atrial fibrillation and flutter (14%), aortic aneurysm (17%), and PAD (8%).

**Fig. 2:**
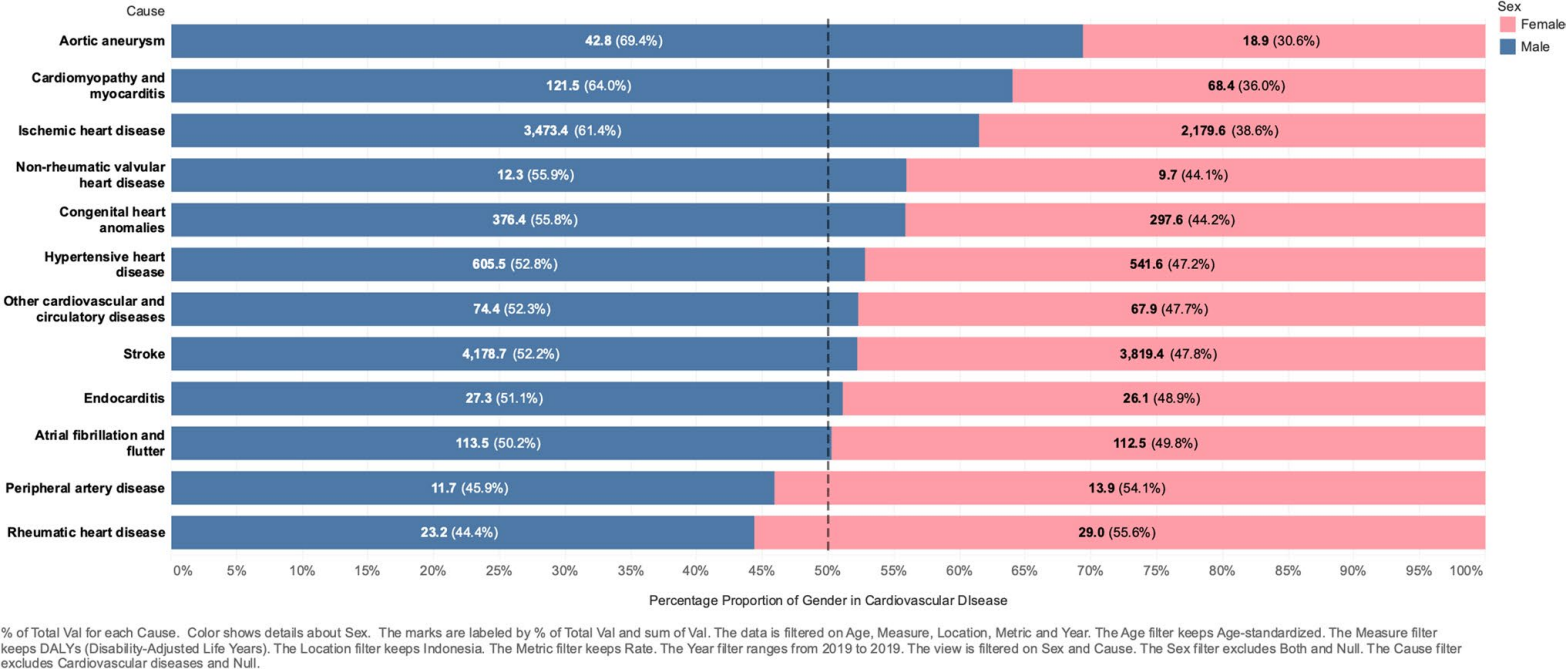
The proportion of gender DALYs rate in all cardiovascular diseases. [The graph shows the DALYs rate, and its percentage compared with all genders in each cardiovascular disease. Blue indicates male, and pink indicates female]. Almost all cardiovascular diseases

As shown (Fig. 2), CVD categories exhibited different proportions between males and females. RHD and PAD dominated females, with 57% and 54% age-standardized DALYs rate (ASDR) proportions, respectively. The most significant DALY proportions in males were for aortic aneurysm, cardiomyopathy, myocarditis, and IHD, with 69.4%, 64%, and 61.4% ASDR proportions, respectively. The remaining diseases were slightly different or similar in terms of gender proportions.

### Provincial-Level Burden

The highest age-standardized DALY rate for total CVD was observed in Bangka Belitung, North Maluku, and South Kalimantan, all more than 10,000 per 100,000 population. During the given period, the ASDR of almost all provinces ranged between 6000 and 10,000 per 100,000 population, except North Kalimantan, at 4084 per 100,000 in 2019, representing the province with the lowest DALYs rate in Indonesia (Table 5).

**Table 5:** All-age DALYs numbers and age-standardized DALYs rates by province and their percentage changes (1990–2019) [one star (*) indicates top three province with biggest percentage increase of age-standardized DALYs rate, two stars (**) indicate the province with biggest percentage decrease]

Disease Sub-Category	All-Age DALYs, No. in Thousands (95% UI)			Age-Standardized DALYs rate (95% UI), per 100 000		
	1990	2019	Change	1990	2019	Change
Aceh	182.4 (138.3–240.9)	309.5 (239.5–387.5)	69.7%	9031.9 (7240.5–11,147.8)	9083.1 (7188.6–10,937.8)	0.6%
Bali**	172.3 (138.8–208.1)	262.6 (202.2–327.7)	52.5%	**8680.2 (7094.2–10,347.4)**	**6054.2 (4684.7–7,499.3)**	− 30.3%**
Bangka Belitung Islands	57.0 (43.5–73.3)	109.1 (85.6–136.2)	91.4%	11,559.2 (9166.9–14,313.0)	10,642.4 (8628.1–12,857.4)	− 7.9%
Banten	388.8 (274.5–539.6)	590.2 (447.9–733.7)	51.8%	9108.0 (7099.4–11,499.9)	8807.2 (6855.8–10,491.2)	− 3.3%
Bengkulu	66.9 (49.5–89.7)	121.5 (94.4–149.4)	81.5%	10,197.5 (8086.2–12,769.2)	8978.9 (7212.2–10,689.4)	− 12.0%
Central Java*	1432.6 (1154.5–1747.1)	2697.0 (2116.5–3241.5)	88.3%	**7270.0 (5985.4–8672.9)**	**7855.9 (6212.4–9352.8)**	8.1%*
Central Kalimantan	65.0 (48.2–83.4)	132.5 (96.9–170.3)	103.8%	9955.6 (7595.9–12,250.7)	8614.5 (6490.8–10,569.4)	− 13.5%
Central Sulawesi	92.9 (68.3–124.9)	200.0 (154.5–253.1)	115.3%	9903.6 (7712.3–12,617.8)	9260.3 (7301.5–11,423.1)	− 6.5%
East Java	1761.0 (1426.9–2188.9)	3031.0 (2435.9–3657.1)	72.1%	7414.8 (6081.9–9046.3)	7563.4 (6123.0–9046.3)	2.0%
East Kalimantan	74.2 (56.0–96.1)	209.5 (156.8–275.3)	182.4%	9642.7 (7753.0–11,887.5)	9269.6 (7237.8–11,647.9)	− 3.9%
East Nusa Tenggara	173.5 (125.1–239.7)	314.8 (241.2–392.8)	81.5%	7889.5 (6116.3–10,166.6)	7912.7 (6120.9–9637.6)	0.3%
Gorontalo	34.4 (25.4–46.9)	79.4 (58.5–103.3)	131.0%	8159.8 (6303.2–10,449.6)	8618.5 (6414.0–10,874.2)	5.6%
Indonesia	9256.5 (7547.4–11,181.5)	17,182.8 (14,038.0–19,948.9)	85.6%	8173.3 (6883.8–9534.0)	8115.7 (6715.2–9254.7)	− 0.7%
Jakarta	328.6 (252.7–425.5)	493.6 (371.9–625.8)	50.2%	8123.0 (6637.0–9885.2)	7486.8 (5794.1–9157.7)	− 7.8%
Jambi**	109.7 (82.6–141.0)	209.6 (162.3–256.9)	91.1%	**11,297.2 (8984.1–13,836.1)**	**8859.3 (6990.5–10,449.0)**	− 21.6%**
Lampung	278.1 (209.5–363.1)	473.6 (368.2–598.6)	70.3%	8629.1 (6954.5–10,544.3)	7572.9 (5961.3–9392.5)	− 12.2%
Maluku	77.7 (54.0–111.0)	110.5 (84.1–138.7)	42.1%	10,730.1 (8185.6–14,046.2)	9570.7 (7475.3–11,765.7)	− 10.8%
North Kalimantan	6.4 (4.0–10.0)	15.8 (11.1–21.9)	147.0%	5141.7 (3808.5–7015.6)	4084.2 (2934.9–5478.8)	− 20.6%
North Maluku	48.8 (35.8–66.0)	81.9 (62.5–104.7)	67.8%	12,653.5 (9958.2–16,150.9)	10,581.8 (8310.0–12,903.9)	− 16.4%
North Sulawesi	103.8 (82.7–130.1)	186.6 (146.0–230.2)	79.7%	8910.3 (7169.6–10,935.5)	8678.7 (6843.2–10,474.5)	− 2.6%
North Sumatra	540.6 (408.2–705.6)	913.8 (709.2–1158.9)	69.0%	9237.8 (7406.0–11,346.1)	8895.5 (7077.7–10,885.5)	− 3.7%
Papua	53.4 (34.7–78.3)	205.9 (146.7–280.8)	285.8%	10,845.6 (8299.1–13,743.4)	10,362.8 (7674.8–13,390.3)	− 4.5%
Riau	113.2 (81.2–151.1)	332.4 (245.4–429.1)	193.7%	9125.6 (7109.6–11,326.8)	7816.0 (5994.3–9549.5)	− 14.4%
Riau Islands**	49.2 (36.6–63.9)	71.0 (53.8–89.2)	44.2%	**10,150.4 (8053.0–12,542.6)**	**7607.7 (5926.4–9168.1)**	− 25.0%**
South Kalimantan*	137.5 (94.8–197.7)	324.0 (244.0–415.1)	135.7%	**8994.0 (6749.1–12,048.9)**	**10,108.8 (7838.1–12,629.6)**	12.4%*
South Sulawesi	316.2 (238.4–425.4)	536.1 (419.1–644.3)	69.5%	7833.4 (6169.0–10,010.1)	7626.2 (6010.5–8975.8)	− 2.6%
South Sumatra	237.2 (178.4–313.3)	475.5 (367.7–595.7)	100.5%	8168.6 (6549.8–10,002.0)	8096.3 (6391.1–9920.8)	− 0.9%
Southeast Sulawesi	86.4 (61.4–118.9)	175.5 (134.0–221.6)	103.2%	11,292.5 (8655.6–14,312.8)	9945.5 (7746.7–12,177.4)	− 11.9%
West Java	1412.8 (1088.6–1830.9)	3079.7 (2404.2–3792.0)	118.0%	8150.8 (6636.2–9905.0)	8145.8 (6445.5–9810.0)	− 0.1%
West Kalimantan	176.7 (132.5–236.4)	282.8 (226.0–346.5)	60.0%	9817.0 (7829.4–12,224.5)	8237.5 (6782.5–9729.2)	− 16.1%
West Nusa Tenggara*	159.5 (100.5–252.5)	325.2 (250.9–397.6)	103.9%	**7364.8 (5147.8–10,630.0)**	**8599.6 (6809.8–10,259.2)**	16.8%*
West Papua	24.9 (17.3–34.9)	45.1 (33.1–59.9)	81.0%	9907.9 (7636.6–12,487.5)	9446.1 (7381.4–11,835.7)	− 4.7%
West Sulawesi	56.2 (39.7–78.9)	94.0 (71.6–119.5)	67.1%	12,656.4 (9698.1–16,281.8)	10,029.0 (7767.6–12,431.4)	− 20.8%
West Sumatra	232.6 (171.8–316.2)	354.0 (277.9–432.7)	52.2%	8468.3 (6520.4–11,060.0)	8174.6 (6499.1–9802.2)	− 3.5%
Yogyakarta	206.1 (169.6–246.6)	338.9 (281.0–404.2)	64.5%	8184.0 (6742.5–9750.7)	8132.2 (6746.5–9682.9)	− 0.6%

The most significant age-standardized DALY rates for total CVDs were recorded in Bangka Belitung, North Maluku, and South Kalimantan, all equating to > 10,000/100,000 population. During the study period, the ASDR of almost all provinces ranged between 6000 and 10,000/100,000 population, except North Kalimantan, which experienced 4084/100,000 people, representing the lowest DALY rates in Indonesia (Table 5). Following underlying CVDs, stroke and IHD contributed the most to DALY rates in Indonesia. This domination was also evidenced in all provinces. The stroke had leading DALY rates in almost all provinces, ranging between 29% and 60% of the total CVD DALYs in each province. In contrast, in two provinces on the same island, Central and South Kalimantan, IHD DALYs rates outnumbered stroke DALYs rates by 29% and 6%, respectively. Finally, hypertensive heart disease also should be addressed due to its DALYs rate percentage, which ranges between 6% and 10%.

Between 1990 and 2019, changes in DALY rates varied by province. West Nusa Tenggara (16.8%), South Kalimantan (12.4%), and Central Java (8.1%) had the highest increases in age-standardized DALY rates. East Java and South Kalimantan provinces experienced the most significant rise in age-standardized DALYs rate, with Aortic Aneurysm showing a notable increase of 56.3% and 49.9%, respectively. In comparison, the most significant contributor to the increase in the age-standardized DALYs rate in the province of West Nusa Tenggara was stroke (48.7%).

The three provinces in Indonesia that experienced the highest reduction in the age-standardized DALYs rate for cardiovascular disease were Bali (− 30.3%), Riau Islands (− 25%), and Jambi (− 21.6%). Rheumatic heart disease and congenital heart anomalies were the contributors to these reductions.

Peripheral artery disease was the primary CVD condition that experienced an increase in the age-standardized DALYs rate in every province in Indonesia, with the highest increases observed in Central Kalimantan (27%), East Kalimantan (24%), and East Java (23%). Other cardiovascular diseases that have experienced an increase in the age-standardized DALYs rate in almost every province were atrial fibrillation and flutter and aortic aneurysms.

We observed increased and decreased CVD rates across Indonesian provinces. The three provinces with the highest DALY ASR increases were West Nusa Tenggara (16.8%), South Kalimantan (12.4%), and Central Java (8.1%). East Java and South Kalimantan provinces observed the most notable increments in Age-Standardized DALYs Rate (ASR) linked explicitly to aortic aneurysms, with notable percentages of 56.3% and 49.9%, respectively. On the other hand, stroke (48.7%) emerged as the primary driver behind the notable rise in Age-Standardized DALYs Rate (ASR) in the West Nusa Tenggara province, compared to other factors. The three Indonesian provinces that experienced the highest reduction in DALYs ASR for CVD were Bali (− 30.3%), Riau Islands (− 25%), and Jambi (− 21.6%). RHD and congenital heart anomalies significantly contributed to reduced DALYs ASR.

**Fig. 3:**
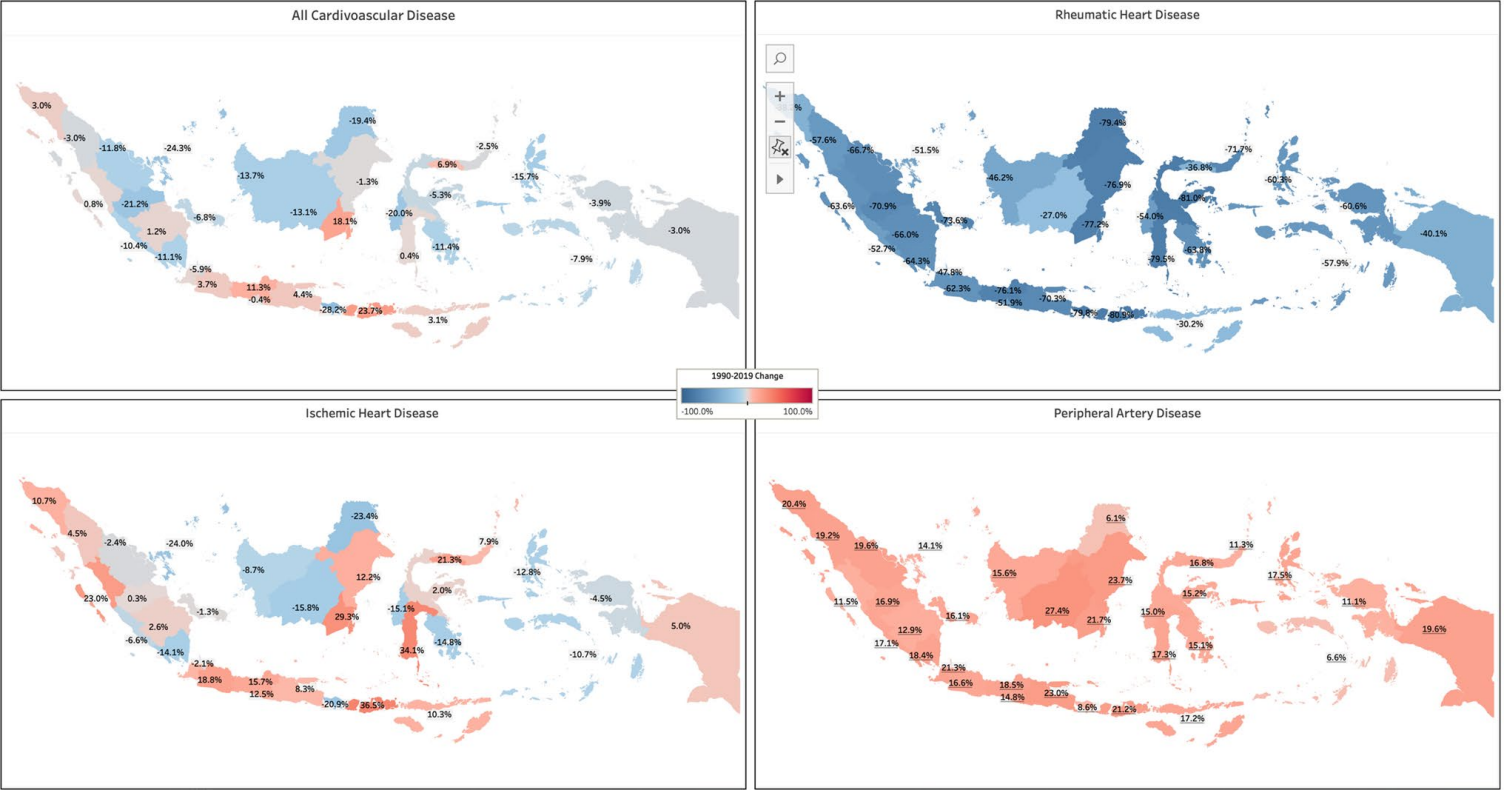
The choropleth of Indonesia’s provinces age-standardized DALY rate of CVD burden change from 1990 to 2019. [Color indicates age-standardized DALYs rate change of cardiovascular disease from 1990 to 2019]

**Fig. 4:**
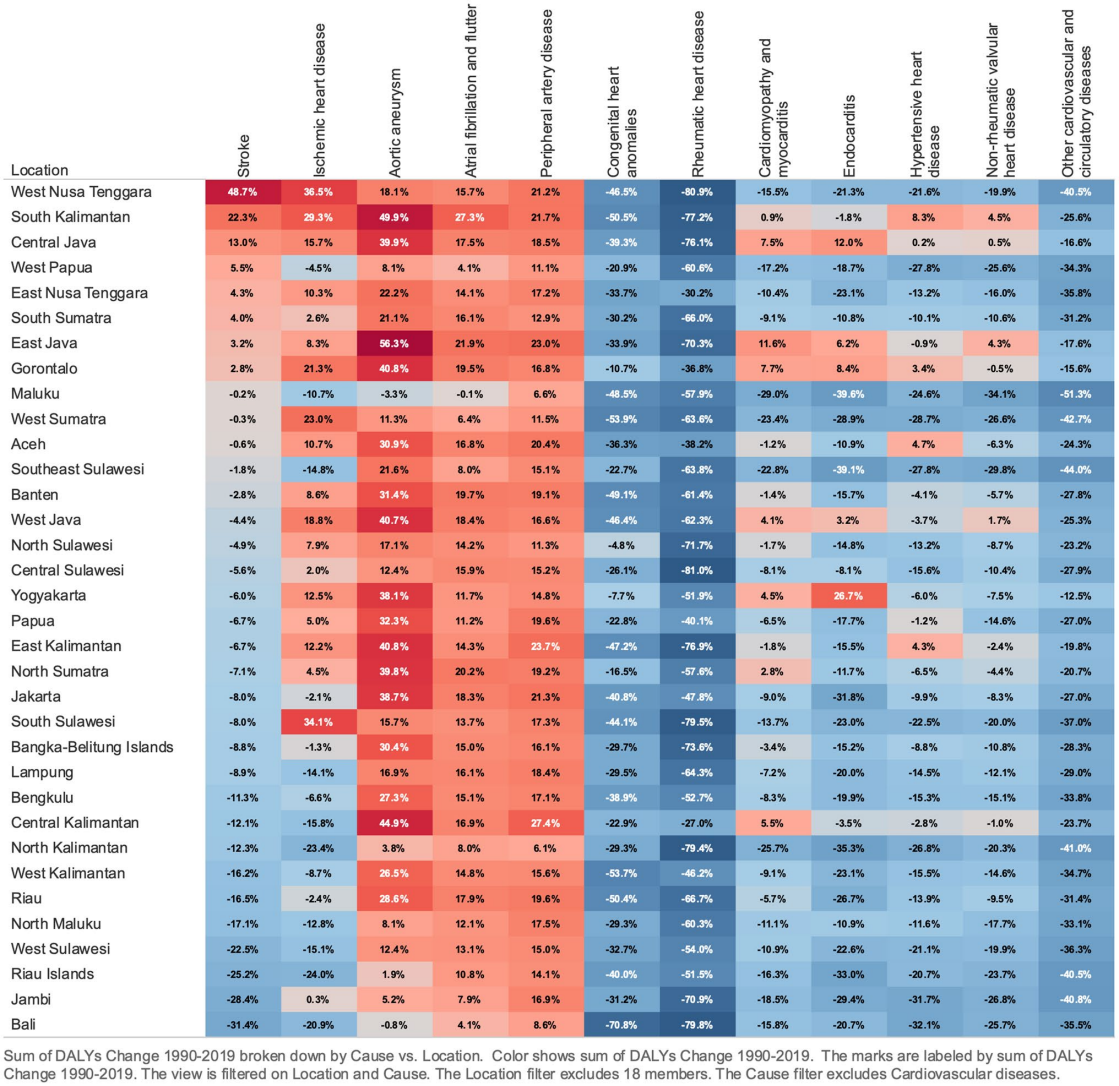
The matrix of progression in age-standardized DALYs rate of cardiovascular disease between 1990 and 2019 in each province. [Row shows 34 province in Indonesia sorted by the percentage change in stroke, column show difference in cardiovascular disease. The color indicates the percentage change between 1990 and 2019, red indicates an increase in DALYs, and blue indicates a decrease]

PAD has experienced increased age-standardized DALY rates in every Indonesian province. The three provinces recording the highest rates were Central Kalimantan (27%), East Kalimantan (24%), and East Java (23%). Other CVDs with increased age-standardized DALY rates in almost every province were atrial fibrillation and flutter and aortic aneurysms (Figs. 3, 4).

### Comparing Indonesia to Other Southeast Asian

**Fig. 5:**
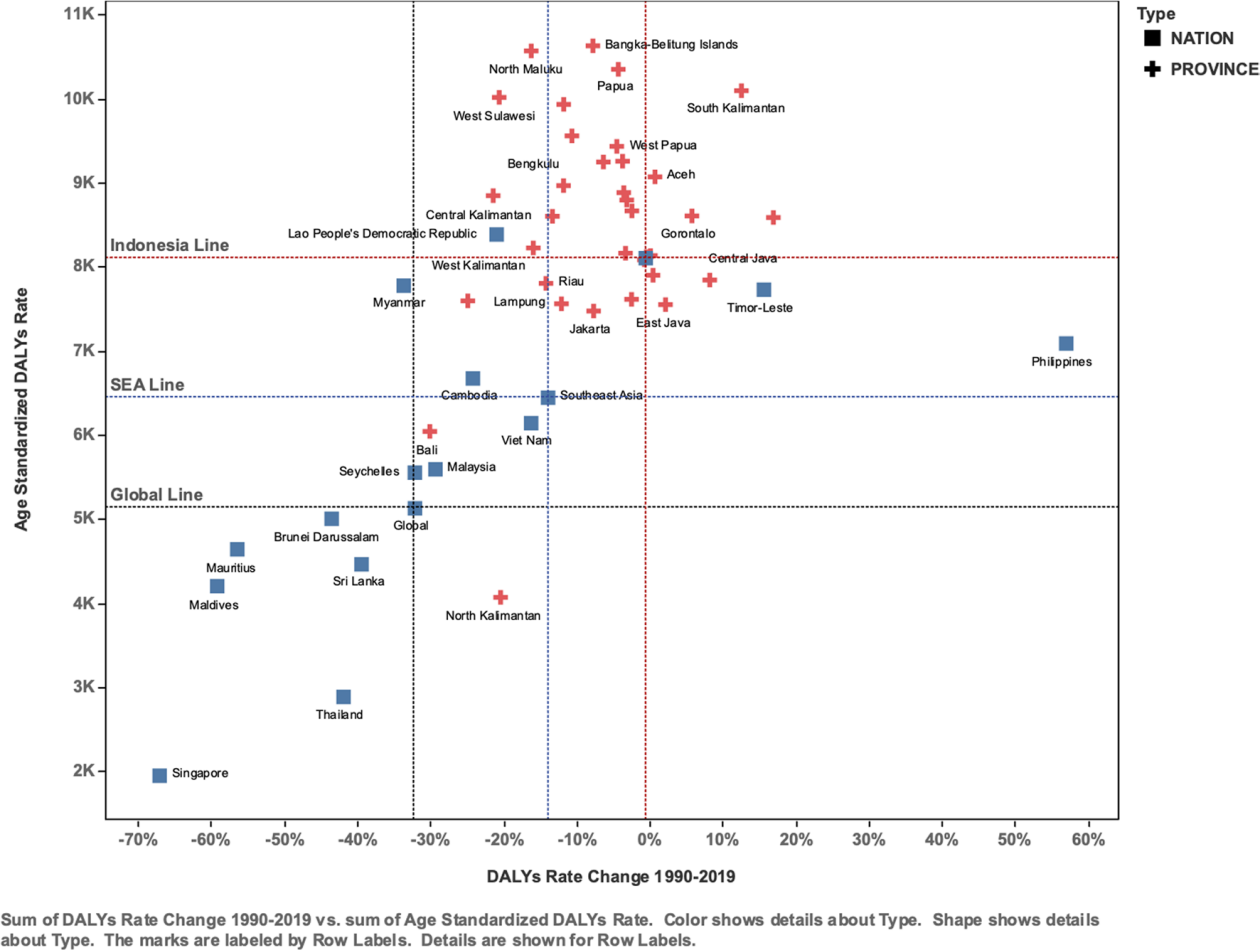
Age-standardized DALYs rate and DALYs rate change of cardiovascular diseases in Indonesia. [*X-axis* shows age-standardized DALYs rate change in 1990–2019, whereas *y*-axis shows age-standardized rate in 2019. Each plus represents provinces in Indonesia, while each box represents Southeast Asia countries. The red vertical line shows a steady DALYs rate change trend. The three horizontal lines constitute the DALYs rate in Indonesia, Southeast Asia, and Global]

By plotting the change in age-standardized Disability-Adjusted Life Years (DALYs) rates from 1990 to 2019 against the absolute DALYs rate in 2019, we categorize Indonesia’s provinces and Southeast Asian countries into four quadrants. The lower left quadrant (small burden size with decreased trends) is the ideal quadrant where common burden conditions occur with decreasing trends. The concern lies with the region in the upper right quadrant, where the burden remains high and is exacerbated by increasing burden trends (Fig. 5). The reference regions were used as a central point of the quadrant; here, we used Indonesia's national average, Southeast Asia's, and the global average.

Red Crosses representing Indonesian provinces are dispersed throughout, indicating a diverse range of CVD burden levels. Provinces depicted in the upper right quadrant are those with a high burden of CVD. Within the figure we see bottom-right quadrant of Indonesia line filled with province with higher income level such as Jakarta and Bali. The blue squares in the graph represent other Southeast Asian countries. Some countries, such as Singapore, Thailand, and Brunei Darussalam, are observed to have lower rates of DALYs, with Singapore's rates notably lower than the global average. Meanwhile, countries like Myanmar and the Lao People's Democratic Republic have higher rates of DALYs, indicating a greater burden of CVD when compared to some of their regional counterparts.

### Risk Factors of Cardiovascular Disease

High systolic blood pressure was the top risk factor for both females and males in both 1990 and 2019, although the DALYs percentage changed slightly over the 30 years. For females, the high body-mass index saw the most significant contribution to DALYs between 1990 and 2019, with a 116% increase. This risk factor saw a massive 169% increase for males over the same period. Air pollution saw a decline in its contribution to DALYs for both genders, with a decrease of 39% for females and 28% for males. There is a dramatic escalation in the significance of high body-mass index. The surge of 116% for females and an even steeper rise of 169% for males is indicative of a growing concern related to obesity and its implications on heart health. This trend might be attributed to lifestyle changes, dietary habits, or societal norms over the past 30 years. Tobacco uses as a cardiovascular risk factor displays notable differences between males and females over 30 years. In 1990, tobacco accounted for 13.6% of male DALYs and 4.5% of females. By 2019, the male contribution slightly rose to 13.9%, maintaining its third ranking, whereas for females, it remained relatively stable at 4.6% but dropped to the eighth rank. These data highlight the consistently higher cardiovascular impact of tobacco on males compared to females.

## Discussion

### Cardiovascular Disease Trends in Indonesia Versus Global Patterns

Indonesia has the hugest share of CVD to total disease burden in Southeast Asia (21.34% of total DALYs) compared to Malaysia (18.88%), the Philippines (16.76%), and Vietnam (20.57%)*.* Contrary to Global and Asian trends, which reduced both Death and DALY rates of cardiovascular disease, Indonesia's increase in age-standardized death rates for CVD by 11.7%, and stagnant in DALY with only − 0.7% change in 30 years. This positions Indonesia fifth worldwide and first in Southeast Asia for CVD deaths, signaling-specific health challenges [18]. According to an Indonesian Ministry of Health report, CVD imposes the most significant cost burden on national health insurance. Based on 2020 data from the National Health Insurance Report, CVD had the highest annual healthcare costs of all diseases and accounted for approximately IDR 9.8 trillion (655 million USD). Additionally, stroke cost burdens ranked third and generated annual healthcare costs of IDR 2.5 trillion (167 million USD) [19, 20].

Estimates from the Global Burden of Disease (GBD) align with findings from previous studies on the cardiovascular disease (CVD) burden in Indonesia, corroborated by WHO data on non-communicable conditions (NCDs) and reports from regional research from Southeast Asia [21–23]. Indonesia exemplifies the trend observed as a middle-income country experiencing an aging population demographic shift, with stroke emerging as the predominant cause of mortality, ahead of ischemic heart disease (IHD). In a comparison across 192 WHO member countries, stroke mortality rates surpassed those of IHD in 74 countries, and stroke disability-adjusted life years (DALYs) exceeded IHD rates in 62 countries. A higher incidence of stroke is notably linked with a higher burden due to a lack of hypertension management in low-to-middle-income countries. Hypertension increase is often undetected and untreated, contributing to higher stroke rates compared to IHD rates during the early stages of demographic and health transitions [24–26].

### Indonesia Pattern in Global Burden Disease

By the rate of prevalence, stroke and peripheral arterial disease are Indonesia's two most common CVDs. PAD is frequently neglected a study of ankle-brachial index examinations in diabetes mellitus patients in Indonesia revealed that 14.5% had lower extremity PAD [27–29]. Another study reported that as many as 42% of chronic kidney disease patients had moderate Ankle-Brachial Index (ABI) results. Moreover, according to PAD guidelines, PAD patients can be placed on a severity spectrum, from asymptomatic to severe symptoms, with > 50% of individuals with PAD being generally asymptomatic. These features could mean that PAD becomes a neglected disease in society. In our results, PAD exhibited significant changes across critical parameters, including prevalence, mortality, and morbidity rates. PAD has emerged as an important public health problem, especially in Indonesia, as it ranks first in Southeast Asia regarding most increased age-standardized DALY rates.

Following the trend of increasing socioeconomic conditions, cardiovascular diseases that are sensitive to socioeconomic conditions and healthcare accessibility [26, 30, 31], congenital heart disease and rheumatic heart disease show significant decreases [30–32]. It has been predicted by the evolution of cardiovascular disease where pestilence and famine, common in low socioeconomic status, will move to degenerative man-made disease [26, 33]. Indonesia's significant change in Gross Domestic Product per capita from 1990 to 2019 ($582 vs. $4151) and reduction in extreme poverty percentage (62.8% vs. 4.4%) play a significant role in RHD and congenital heart disease reduction [34].

In general, CVD Age-Standardized DALYs Rate (ASDR) has remained stagnant for approximately 30 years (− 0.7% change). This contrasts with significant CVD ASDR decreases in global and Southeast Asia rates, indicating that Indonesia bears stubbornly high burden rates from increased premature mortality and years of living with disability [12, 35]. Although decreased DALY rates were recorded for RHD and CHD, this was balanced by DALY gains in non-communicable CVD rates. This is also shown by the stagnancy in progress of reducing premature death from non-communicable diseases in Indonesia. Several factors have contributed to this stagnancy, including CVD risk factors, primary and secondary prevention measures, socioeconomic status, health infrastructure, and a lack of health workers [36–39].

### Gender Difference

**Fig. 6:**
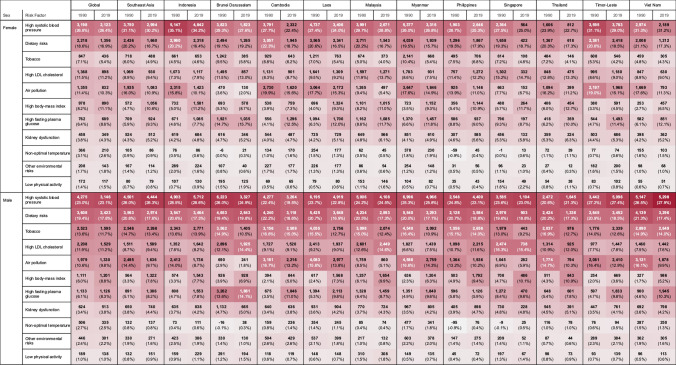
Risk factors contributing to age-standardized cardiovascular disease DALYs rates in Southeast Asian countries. [Numbers in brackets indicate the percentage contribution of each risk factor to the total risk factors for each region and sex; red color indicates a higher percentage]

Despite an overall rise in mortality and stagnant DALYs, there is a marked gender disparity in these trends. CVD mortality rates have increased by 20.3% in men compared to 3.6% in women, leading to a notable difference (357 vs. 416 per 100,000). The reported sex differences in CHD and stroke mortality align with studies showing men generally have higher mortality rates [35, 40]. The findings on sex differences in CVD risk from previous studies have been mixed, with varying results on which gender faces a higher risk. Differences in both behavior and healthcare utilization may influence the observed disparities between genders. Regarding behavioral risk factors, as illustrated in Fig. 6, the burden of CVD attributable to smoking show significantly higher in males, being up to 4.24 times greater than in females (2771 vs. 653). This further evidenced by recent study from WHO. Indonesia overall tobacco usage rates: 34.5% of the adult population (70.2 million adults) currently uses tobacco products (smoking, smokeless, or heated tobacco products), with a significant gender divide where 65.5% of men and only 3.3% of women are tobacco users [41].

Recent study also indicates that men are less likely to seek primary care compared to their female counterparts. This behavioral pattern may contribute to the observed disparities in health outcomes between genders [42, 43]. The lower number of men utilizing primary care services can be attributed to accessibility challenges, particularly for working-age males whose job schedules, predominantly in the morning, conflict with primary care operating hours. Compliance and adherence are important factors of chronic CVD management, and policymaker needs to adjust the preventive approach of primary care (i.e., add evening or weekend hours of primary care) [44, 45].

### Variation in Provincial Level

Despite the stagnancies of total DALY, Indonesia has made commendable strides in curbing mortality and morbidity rates associated with congenital heart disease and rheumatic heart disease. This transformation signals a shift from infectious and maternal issues to chronic ailments predominantly observed in the elderly. This evolution can be attributed to enhanced child and maternal healthcare services. Simultaneously, Indonesia is undergoing a demographic transition with an increasing elderly population. Same pattern is shown in global and regional [46–50]. This trend is particularly evident in provinces with significant aging populations like West Nusa Tenggara and Central Java. Here, the disease pattern largely mirrors the national trend, with a notable exception for ailments like stroke, ischemic disease, and other chronic CVDs. These particular diseases have substantially increased in these provinces, indicating region-specific health challenges amidst the broader national progress [51].

### Increase in Risk Factor of Cardiovascular Disease

The shifting CVD burden has been attributed to multiple factors, including economic, behavioral, demographic, health workforce, and health infrastructure factors. In terms of economic factors, the economy has grown over the last 30 years and caused a shift in CVD burden from a lower poorer economy (RHD) to a CVD burden in workers with increased incomes (IHD) [26, 27]. Regarding behavioral factors, high blood pressure and smoking are standard in Indonesia and are the primary causes of increased DALY rates. Fail on risk reduction is still the concern in Indonesia, where healthcare fails to make hypertension patients still on the cascade of care, and only 15% of patients with hypertension routinely control to primary healthcare [38, 52–55].

The GBD risk factor result has shown that hypertension is the leading cause of CVD, accounting for 36%–42% of all strokes and 20%–25% of all IHDs. Additionally, smoking in males led to a large proportion of IHD (25%) and stroke (17%). High blood cholesterol is also a significant risk factor for 17.8%–19% of IHD events and 10% of all ischemic strokes [52, 56–58]. Southeast Asia has one of the highest total cholesterol increases, with four countries implicated: Indonesia, Thailand, Malaysia, and Cambodia. Indonesia is one of the five countries with the highest number of adults with diabetes, accounting for 48% of all diabetes patients in the world [59]. It is also in the top ten countries with the highest number of deaths from high blood sugar [60, 61].

### The Economic Impact of CVD Burden

The economic impact of cardiovascular disease (CVD) can be understood at the micro- and macro-levels [62]. On the macro-level, the economic burden is seen in the loss of productive years within the population. This includes premature deaths, quantified as Years of Life Lost (YLL), and the disability caused by the disease, measured as Years Lived with Disability (YLD). Reducing premature mortality due to CVD would improve health outcomes and maximize human capital productivity over an extended period [62–65]. In the specific context of Indonesia, which is experiencing economic growth, the aging population with a sustained burden of CVD presents a potential challenge. If the burden of CVD remains unaddressed, it could significantly hinder Indonesia's economic progress by increasing the cost of illness and reducing the maximum capabilities of human capital.

On the micro-level, it involves individuals' catastrophic health expenditure (CHE) in treating CVD. Studies have shown that NCD CHE increased with the NCD share of DALYs, especially for vulnerable populations [65, 66]. A Study from Vietnam explains that the burden is not only at the individual level but also at the household level. A family with one member older than 60 years old with CVD is likely to suffer and reduce the economic capabilities of all family members. This further may threaten Indonesia poverty reduction efforts [64].

### The Limitations

While our research presents a broad view of the burden of cardiovascular diseases (CVDs) in Indonesia, it is essential to acknowledge its limitations. Notably, several references underscore the limitations of utilizing GBD data. It may have inherent biases and inaccuracies. Inaccuracy was common in estimating dates, and uncertainty measurements were needed. The 95% uncertainty interval (UI) is provided in the table. The range of uncertainty needs to be considered if it is too wide. This study also could not answer the disparities yet due to not investigating the socioeconomic status. However, given the comprehensiveness of GBD data and the absence of frailty estimates in many countries, this approach remains valuable for global frailty monitoring and comparison. As frailty gains recognition as a significant public health challenge, comparative estimates like those provided by GBD are increasingly essential for policymakers and healthcare planners. These estimates are particularly crucial in contexts where formal frailty assessment is unfeasible. They support effective policymaking and resource allocation, addressing public health challenges more specifically.

### Recommendations for Future Research and Policymaker

Given the insights and limitations identified, future research has several directions. A deeper exploration into the underlying causes of the identified trends, especially in provinces like West Nusa Tenggara and Central Java, would be beneficial. Furthermore, there is a need for more comprehensive studies focusing on the gender disparities in CVD mortality and morbidity rates. Investigating the behavioral, socioeconomic, and cultural factors contributing to these disparities will provide a richer understanding.

Indonesia is shifting toward chronic care management to fight cardiovascular diseases (CVD), impacted by high systolic blood pressure, nutrition, and smoking. Our analysis shows they are key CVD factors. Indonesia has developed focused techniques to aggressively identify at-risk persons. Integrated NCD Community Care programs like Posbindu and Prolanis are implemented. However, make patient compliance to care still difficult. This emphasizes the necessity for evaluations to enhance healthcare delivery and patient commitment in chronic care. To reduce CVD risks, population-level interventions including taxes on high-salt and high-sugar items, tobacco tax and cessation programs, and infrastructure that promote physical activity are essential.

## Conclusions

This study highlights Indonesia's burden change of cardiovascular diseases (CVD) over the past three decades, which contrasts with global trends. While RHD dramatically reduced, it highlights stroke and IHD as the primary contributors to the increase in CVD-related deaths and DALYs. Peripheral artery disease, often overlooked, also shows worrying trends. While Global Burden of Disease (GBD) estimates provide a broad view, the limitations inherent in this data source underscore the need for enhanced capabilities in Indonesia's national and provincial CVD registries and assessments to further become the basis of intervention.

### Supplementary Information

Below is the link to the electronic supplementary material.

Supplementary file 1 (XLSX 19 KB)

## Data Availability

The datasets generated and/or analyzed during the current study are available from the corresponding author on reasonable request. Restrictions apply to the availability of these data, which were used under license for this study, and so are not publicly available.

## Abbreviations

ABI: Ankle-brachial index
AF: Atrial fibrillation and flutter
ASDR: Age-standardized DALYs rate
ASPR: Age-standardized prevalence rate
ASR: Age-standardized rate
CHD: Congenital heart disease
CODEm: Cause of Death Ensemble model
CVD: Cardiovascular disease
DALYs: Disability-adjusted life years
EAPC: Estimated annual percentage change
GBD: Global Burden of Disease
IHD: Ischemic heart disease
NCD: Non-communicable diseases
PAD: Peripheral artery disease
RHD: Rheumatic heart disease
ST-GPR: Spatiotemporal Gaussian process regression
UI: Uncertainty interval
WHO: World Health Organization
YLDs: Years lived with disability
YLLs: Years of life lost
