# Correction: The 30 Years of Shifting in The Indonesian Cardiovascular Burden—Analysis of The Global Burden of Disease Study

**DOI:** 10.1007/s44197-024-00280-y

**Published:** 2024-08-05

**Authors:** Farizal Rizky Muharram, Chaq El Chaq Zamzam Multazam, Ali Mustofa, Wigaviola Socha, Andrianto, Santi Martini, Leopold Aminde, Chung Yi-Li

**Affiliations:** 1 Global Health and Social Medicine Department, Harvard Medical School, Boston, MA USA; 2 Cardiology and Respiratory Department, Imperial College London, London, UK; 3 Department of Cardiology and Vascular Medicine, Faculty of Medicine, Airlangga University, Soetomo General Hospital, Surabaya, Indonesia; 4 Faculty of Public Health, Airlangga University, Surabaya, Indonesia; 5 Population Health and Research Methods Department, School of Medicine and Dentistry, Griffith University, Brisbane, QLD Australia; 6 Institute of Public Health, National Cheng Kung University, Tainan City, Taiwan

**Correction: Journal of Epidemiology and Global Health (2024) 14:193-212**

10.1007/s44197-024-00187-8

When this article was published, a mismatch between the Results-section of the Abstract and the text was noted. Instead of “CVD deaths have doubled from 278 million in 1990 to 651 million in 2019”there should be “All-age CVD deaths more than doubled from 292 thousand (95% UI: 246 to 339 thousand) in 1990 and increased to 659 thousand (95% UI: 542 to 747 thousand) in 2019”.

The Original Article has been corrected.

